# Association between albuminuria and retinal microvascular parameters measured with swept-source optical coherence tomography angiography in patients with diabetic retinopathy

**DOI:** 10.1371/journal.pone.0295768

**Published:** 2024-03-06

**Authors:** Jin Sug Kim, Eung Suk Kim, Hyeon Seok Hwang, Kyung Hwan Jeong, Seung-Young Yu, Kiyoung Kim

**Affiliations:** 1 Division of Nephrology, Department of Internal Medicine, Kyung Hee University Hospital, Kyung Hee University, Seoul, South Korea; 2 Department of Ophthalmology, Kyung Hee University Hospital, Kyung Hee University, Seoul, South Korea; Cairo University Kasr Alainy Faculty of Medicine, EGYPT

## Abstract

**Purpose:**

To evaluate the relationship between urine albumin excretion (UAE) and retinal microvascular parameters assessed using swept-source optical coherence tomography angiography (SS-OCTA) in patients with diabetic retinopathy (DR).

**Methods:**

This retrospective cross-sectional study included 180 patients with diabetes and 50 age-matched controls. Patients with diabetes were grouped according to the five-stage DR severity, combined with the presence of albuminuria. All subjects underwent 12×12mm^2^ field SS-OCTA. The foveal avascular zone metrics, vessel density, and capillary nonperfusion area (NPA) were quantified using a semi-automatic software algorithm on three different rectangular fields (3×3 mm^2^, 6×6 mm^2^, and 10×10 mm^2^). The correlations between albuminuria and the four OCTA parameters were analyzed.

**Results:**

A total of 105 subjects had normal UAE, and 75 subjects had albuminuria. Of the 102 subjects whose DR severity was higher than mild non-proliferative DR (NPDR), capillary NPA on the 3×3 mm^2^, 6×6 mm^2^, and 10×10 mm^2^ fields was significantly larger in the albuminuria group. None of the OCTA parameters were significantly different between the two groups in subjects with mild NPDR or without DR. Multiple logistic regression analysis showed that an increase in NPA in the 6×6 mm^2^ and 10×10 mm^2^ fields was a significant risk factor for the presence of albuminuria (odds ratio = 1.92 and 1.35).

**Conclusion:**

An increase in capillary NPA was independently associated with albuminuria in patients with clinically significant DR levels. SS-OCTA imaging can be a useful marker for the early detection of diabetic nephropathy.

## Introduction

Both diabetic retinopathy (DR) and nephropathy are chronic microvascular complications commonly observed in individuals with diabetes [1]. Diabetic nephropathy stands as the foremost cause of end-stage renal failure, while retinopathy is the leading cause of blindness in patients with diabetes.

These diabetes-specific microvascular disorders affecting the retina and glomerulus share similar pathophysiological characteristics. Diabetic nephropathy is characterized by increased urinary albumin excretion and a decreased glomerular filtration rate [2]. Within the glomerulus, renal insufficiency and urinary protein loss coincide with widespread capillary occlusion and loss glomerular podocyte [3]. Consequently, microalbuminuria has been recognized as an early indicator of endothelial dysfunction, playing a role in the pathogenesis of various systemic conditions [4, 5]. Extensive research has detailed the involvement of retinal vascular dysfunction in the pathophysiology of DR. In the retina, diabetes induces the death of Müller and ganglion cells, and pericytes and endothelial cells, resulting in retinal ischemia and initiating a cascade of events that culminate in vasculopathy [6].

Numerous clinical investigations have reported a significant association between retinopathy and nephropathy in patients with diabetes. There is a significantly increased risk of DR for subjects with microalbuminuria [7, 8], and diabetic patients with albuminuria frequently present with vision-threatening DR [9]. Nagaoka et al. employed doppler velocimetry to identify reduced retinal blood flow in early diabetic kidney disease [10]. Lim et al. found that changes in retinal vascular geometry assessed through fundus photography are associated with renal dysfunction [11]. Recent advances in spectral-domain optical coherence tomography (SD-OCT) have unveiled structural changes in the neuroretinal layer that correlate with diabetic nephropathy and declining renal function [12]. Additionally, the advent of swept-source optical coherence tomography angiography (SS-OCTA), offering superior axial resolution and rapid contrast-free acquisition of retinal vasculature, has enabled accurate assessment of microvascular parameters within individual retinal layers. Even prior to clinically detectable DR, alterations in macular OCTA parameters, such as enlargement of the foveal avascular zone (FAZ), increased capillary nonperfusion area (NPA), and reduced capillary density, have been documented in diabetic eyes [13, 14].

Although previous studies have focused on the relationship between renal function and vascular changes in DR, microvascular components have not been fully evaluated. Until recently, only a few studies have measured OCTA parameters in diabetic nephropathy, and the current study aimed to describe the clinical association between albuminuria and SS-OCTA parameters across different stages of DR.

## Methods

A total of 180 patients diagnosed with type 2 diabetes mellitus without renal impairment (estimated glomerular filtration rate ≥ 60 mL/min per 1.73 m^2^) and 50 age-matched healthy controls between September 2018 and 2020 were enrolled in the current study. Data related to the patient’s age, sex, diabetes duration, systemic variables, and detailed ophthalmic evaluation at their first visit were collected from medical records. This study was approved by the Institutional Review Board (IRB) of Kyung Hee University Medical Center (2022-10-059). The need for written informed consent was waived due to retrospective study and use of anonymized data. This study was conducted in accordance with the tenets of the Declaration of Helsinki.

Subjects with diabetes were divided into two groups based on the presence of albuminuria. The albumin-to-creatinine ratio (ACR) in a random spot urine collection was used to diagnose albuminuria. Albuminuria was defined as an ACR of 30 mg/g or greater. Data on age, sex, height, weight, duration of diabetes, presence of hypertension, and laboratory test results, including glycated hemoglobin (HbA1c), serum albumin, and creatinine, were also collected. The estimated glomerular filtration rate (eGFR) was calculated using the Chronic Kidney Disease Epidemiology Collaboration (CKD-EPI) equation.

All patients were diagnosed with treatment-naïve DR and underwent color fundus photography and SS-OCTA imaging. DR severity was graded by two graders by consensus based on the auto-montage images of fundus photographs. The DR grade was defined using the Early Treatment Diabetic Retinopathy Study standard grading protocol as no DR (grade 0), mild non-proliferative DR (NPDR), moderate NPDR, severe NPDR, and proliferative DR (PDR). All SS-OCTA scans were acquired over a 12×12 mm^2^ field using the PLEX^®^ Elite 9000 (ZEISS, Dublin, CA, USA) device, which uses a wavelength of 1050 nm and operates at 100,000 A-scans per second. This instrument has an axial resolution of approximately 5 μm and a lateral resolution of 14 μm. The 12×12 mm^2^ scans used 500 A-scans per B-scan at 500 B-scan positions, resulting in an A-scan and B-scan separation of 24 μm [15]. Co-registered structural enface OCT images were acquired using built-in software. The exclusion criteria included a history of laser photocoagulation, intravitreal injection, vitreous hemorrhage, and cataracts that limited the ability to obtain an SS-OCTA scan with a signal strength index lower than seven.

**Fig 1:**
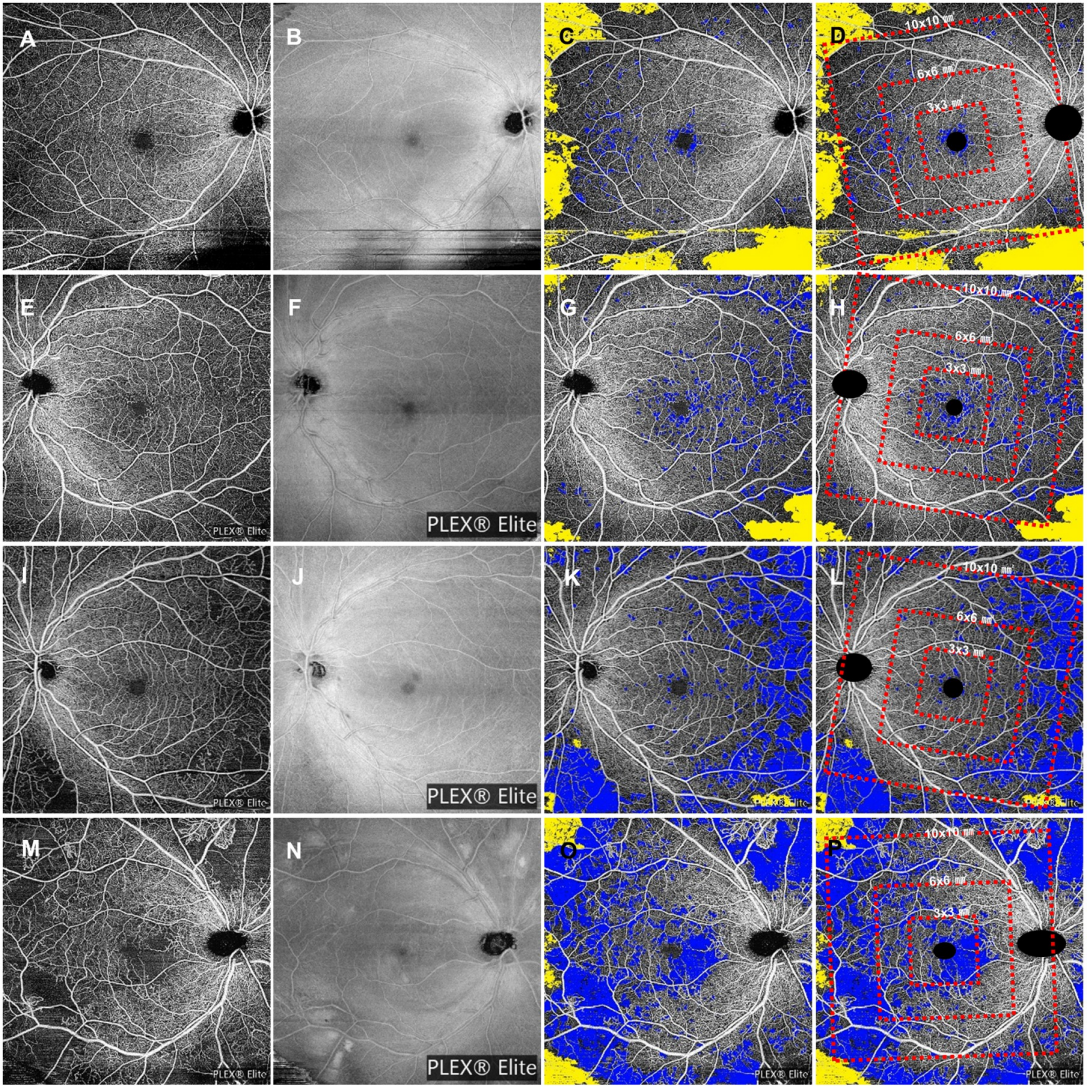
Representative SS-OCTA images for the analysis of the capillary nonperfusion area in patients with different grades of DR. (A): 12x12mm SS-OCTA images of the total retinal slab in patients with mild NPDR. (B): Co-registered structural en face OCT images for the exclusion of low-signal artifacts. (C) The signal loss area due to low-signal artifacts was manually delineated using the color yellow and excluded from the calculation of the nonperfusion area. The true nonperfusion area was detected with semi-automatic software and marked in blue. (D) Three different regions of interest were overlayed in the final images. The angle of the square region was defined as the parallel to the optic disc-fovea line. Rectangular images covering 3x3 mm^2^, 6x6 mm^2^, and 10x10 mm^2^ were analyzed. (E-H): SS-OCTA images with the nonperfusion and artifacts areas marked in a patient with moderate NPDR. (I-L): Representative SS-OCTA images with the nonperfusion and artifacts areas marked in a patient with severe NPDR. (M-P): Representative SS-OCTA images with the nonperfusion and artifacts areas marked in a patient with PDR.

SS-OCTA parameters were measured using custom MATLAB software (MathWorks, Inc., Natick, MA) based on a total retinal slab of a 12×12 mm^2^ image. Three different sizes of cropped images covering 3×3 mm^2^, 6×6 mm^2^, and 10×10 mm^2^ regions centered on the fovea were selected after excluding the disc and large retinal vessels. The NPA was defined as a continuous region lacking microvasculature through the utilization of a sliding square kernel-based thresholding method. The degree of perfusion and the presence of microvasculature at each pixel location on the SS-OCTA image were assessed by computing the average signal value within a square kernel, sized at 17 x 17 pixels, centered on the respective pixel. The threshold value for binarizing the NPA was determined following the method previously outlined by Kim et al. [16], which utilized 250 pixels on the same SS-OCTA instrument. Vessel density (VD) was quantified utilizing the global mean binarization threshold. As previously mentioned, [16] major vessels were excluded, and the measured area was binarized based on the mean intensity of the SS-OCTA signal. VD was determined by counting the number of pixels within a specified area and converting this count into VD. Finally, boundaries delineating the FAZ were manually outlined on a superficial 3 x 3 mm^2^ scan slab. The area and circularity of the FAZ were computed using Image J, a software tool developed by the National Institutes of Health, Bethesda, MD, USA. The signal reduction artifacts were recognized using the structural enface OCT images and manually excluded by a retinal specialist. Then, the signal loss area due to artifacts was excluded from the calculation of the capillary NPA. In Fig 1, the representative SS-OCTA images are marked with capillary NPA and low-signal artifacts, and three different regions of interest are overlaid.

Statistical analysis was performed using SPSS version 18.0 (SPSS, Chicago, IL, USA). The results of the continuous variables are shown as the mean values and standard deviation (SD) or the number and percentage of patients. Comparisons between groups were performed using an analysis of variance, followed by Tukey’s post-hoc analysis to correct for multiple comparisons. Logistic regression analysis was used to determine the association between OCTA parameters and the presence of albuminuria. A P-value less than 0.05 was considered significant.

## Results

The clinical characteristics of the patients with diabetes enrolled in this study are summarized in Table 1. Of the 180 subjects with diabetes, 31 had no DR, 25 had mild NPDR, 49 had moderate NPDR, 52 had severe DR, and 23 had PDR. Patients were divided into two groups according to the UAE level. Albuminuria was present in 75 patients (41.7%), and normal UAE was observed in 105 patients (58.3%). There was a significant difference in the HbA1c levels between patients with albuminuria and those with normal UAE (P = 0.008); no significant differences were observed between the two groups for the other variables except the renal profile, such as age, sex, mean arterial pressure, and diabetes duration. There was a significant difference in the distribution of DR grades between the two groups. The percentage of patients with DR grades lower than moderate NPDR (no DR and mild NPDR) was 16.0% (12/75) in the albuminuria group and 41.9% (44/105) in the normal UAE group.

**Table 1:** Characteristics of the study population.

	Diabetes without albuminuria	Diabetes with albuminuria	P-value
Number, n	105	75	
Age, yrs	57.3±10	55.5±10	0.672
Male/Female	1.4	1.35	0.833
Mean arterial pressure, mmHg	91.9±11.1	95.8±13.2	0.315
Duration of DM, yrs	8.7±6.9	9.8±7.5	0.625
Urinary albumin excretion, mg/L	11.4±6.9	631.7±990	0.001*
eGFR, ml/min/1.73m^2^	105±31.1	87.2±46.9	0.042
HbA1c, %	7.2±1.2	8.1±2.1	0.008*
BCVA, logMAR	0.96±0.09	0.90±0.15	0.155
Grade of DR, n			0.001‡
No DR	21	10	
Mild NPDR	23	2	
Moderate NPDR	28	21	
Severe NPDR	26	26	
PDR	7	16	

BCVA = best-corrected visual acuity; DM = diabetes mellitus; DR = diabetic retinopathy; eGFR = estimated glomerular filtration rate; NPDR = non-proliferative diabetic retinopathy; PDR = proliferative diabetic retinopathy.

*Analysis of variance, followed by Tukey’s post-hoc analysis,

^‡^ Chi-square test.

There were no significant differences in the OCTA parameters between the albuminuria and normal UAE groups. We excluded patients without DR and those with mild NPDR to adjust for potential effects from the different distributions of DR grades between the two groups. The differences in the OCTA parameters between 124 patients with DR grades higher than mild NPDR were compared to controls (Table 2). The FAZ parameters were not significantly different between the albuminuria and normal UAE groups. The mean NPA in the albuminuria group was significantly larger in the 6x6 mm^2^ and 10x10 mm^2^ fields compared to the normal UAE group (1.67 and 21.8 vs. 0.76 and 13.5, P = 0.001). The mean VD showed a decreasing trend in the albuminuria group compared to the normal UAE group in the 3×3, 6×6, and 10×10 mm^2^ fields, but the difference was not significant.

**Table 2:** Comparison of the OCTA parameters in patients with DR grades higher than those with mild NPDR according to the presence of albuminuria.

	Controls	Diabetes without albuminuria	Diabetes with albuminuria	P-value
Number (n)	50	61	63	
Grade of DR (n)				0.167
Moderate NPDR		26	14	
Severe NPDR		24	20	
PDR		6	10	
FAZ area (mm^2^)		0.42±0.13	0.42±0.24	0.450
FAZ circularity		0.56±0.12	0.54±0.11	0.113
Nonperfusion area (3x3 mm^2^)		0.45±0.26	0.65±0.7	0.083
Nonperfusion area (6x6 mm^2^)		0.76±0.54	1.67±2.3	0.001*
Nonperfusion area (10x10 mm^2^)		13.5±7.7	21.8±20	0.001*
Vessel density (3x3 mm^2^)		46.3±5.2	44.6±5.5	0.965
Vessel density (6x6 mm^2^)		52.5±3.9	51.1±4.1	0.701
Vessel density (10x10 mm^2^)		48.9±2.6	46.8±3.1	0.142

DR = diabetic retinopathy; FAZ = foveal vascular zone; NPDR = non-proliferative diabetic retinopathy; PDR = proliferative diabetic retinopathy.

*Analysis of variance followed by Tukey’s post-hoc analysis.

Table 3 presents the results of the multiple logistic regression analysis to determine the association between albuminuria and capillary NPA on OCTA images with different fields of view. An increase in NPA in the 6×6mm^2^ and 10×10 mm^2^ fields was significantly associated with an increased risk of albuminuria after controlling for other factors such as age, sex, diabetes duration, hypertension, and HbA1c.

**Table 3:** Multiple regression analysis was performed to identify the risk factor profile of capillary nonperfusion areas in different fields of view for the presence of albuminuria.

	Odds ratio	95% CI	P-value
Nonperfusion area			
3x3 mm^2^ scan	3.509	0.970–12.701	0.056
6x6 mm^2^ scan	1.921	1.053–3.808	0.029*
10x10 mm^2^ scan	1.347	1.021–2.726	0.041*

CI = Confidence Interval

*Adjustment for age, sex, diabetes duration, hypertension, and HbA1c level.

When the optimal cut off point was estimated for the prediction of albuminuria, cut-off value of NPA in 10×10 mm^2^ fields was 4.27 corresponding to the 81% sensitivity and 64% specificity. The mean of excluded area for the FAZ and optic disc was 1.51.”

## Discussion

In this study, we utilized 12×12 mm^2^ SS-OCTA images to assess retinal microvascular changes, particularly capillary NPA and VD, in different stages of DR. Additionally, we explored the relationship between albuminuria and OCTA parameters across various image sizes. Notably, our findings highlight that larger field of view OCTA scans may be more effective in detecting early microvascular changes in DR.

The simultaneous advancement of diabetic retinopathy and nephropathy suggests shared underlying mechanisms, such as endothelial dysfunction [17]. Prolonged high blood sugar levels play a central role in initiating microvascular complications in diabetes [18]. This leads to damage to the endothelial cells, thickening of the basement membrane, aggregation of platelets, disruption of the retinal barrier, and adhesion of white blood cells to retinal capillaries [19]. In diabetes, the presence of chronic hypoxia triggers the release of angiogenic growth factors, promoting the formation of new blood vessels in the retina and the development of proliferative DR. In diabetic nephropathy, hyperglycemia also induces inflammatory cytokines, advanced glycation end products, reactive oxygen species, and vascular endothelial growth factor. These factors contribute to endothelial damage in the renal capillaries, glomerular hyperfiltration, and the presence of microalbuminuria [20, 21]. Advanced nephropathy is characterized by clinical albuminuria, progressive decline in GFR, and elevated blood pressure [22].

Microalbuminuria, which alters the permeability of the endothelial layer, allows lipids to enter the vessel walls, contributing to systemic atherosclerotic changes, including those observed in the retinal artery [23]. Previous studies have reported the impact of microalbuminuria on the diabetic retina. Farias et al. observed a decrease in subfoveal choroidal thickness during the microalbuminuria stage of diabetic patients, indicating that early microvascular choroidal changes before clinically visible DR may be due to atherosclerotic effects of microalbuminuria [24]. Atilgan et al. also found a significant decrease in parafoveal retinal thickness in patients with mild DR and microalbuminuria compared to those without DR and microalbuminuria [25].

It is well established that diabetic nephropathy, characterized by progressive microvascular alterations, can be detected early through the presence of albuminuria. Chen et al. demonstrated that microalbuminuria is a better predictive parameter than moderately reduced estimated GFR in determining retinal outcomes in patients with type 2 diabetes [26]. Microalbuminuria, as a marker of generalized endothelial damage, can increase the permeability of small retinal capillaries and appears to be associated with decreased retinal microcirculation in early-stage DR [10]. Surawatsatien N et al. described significant reduction of VD in late diabetic nephropathy group, but we could not find significant difference in VD value according to the albuminuria [27]. Liu et al. also found a strong correlation between diabetic macular edema and high levels of albuminuria [28]. Selective thinning of the neuroretinal layer has been observed in diabetic patients with microalbuminuria and no clinically visible retinopathy, suggesting that thinning of the inner retinal layer may precede microvascular changes in early-stage DR [12, 29]. Consistent with this finding, the current study revealed that the correlation between retinal microvascular parameters and albuminuria was significant in subjects with more severe than mild NPDR but not in those with mild NPDR or without DR. This result could be explained by the fact that mGCIPL thinning precedes microvascular impairment in the early-stage of DR. Therefore, an increase in capillary NPA was independently associated with albuminuria in subjects whose DR grade was higher than mild NPDR. Furthermore, microalbuminuria has been identified as a marker for the risk of developing DR [7], and Manaviat et al. demonstrated a correlation between microalbuminuria and proliferative DR [7, 30]. Our results suggest that vascular insufficiency may be involved in the shared pathogenesis of diabetic complications, indicating an association between impaired kidney function, retinal neurodegeneration, and reduced microcirculation. Similar to the pathogenesis of ischemia in the diabetic retina, the progression of diabetic nephropathy is ultimately linked to capillary nonperfusion, leading to the death of podocyte cells, angiogenesis in the glomerular capillaries, and loss of capillaries in the glomerulus and interstitium [31, 32]. Advanced stages of nephropathy are accompanied by changes in glomerular histology and increased excretion of proteins [33].

Capillary occlusion leading to retinal ischemia manifests as a flow void signal, indicating retinal NPA in OCTA images [34]. Previous studies have demonstrated a correlation between more extensive NPA on OCTA and higher DR severity [16, 35]. These findings suggest that quantifying NPA can serve as a novel OCTA biomarker for early detection and monitoring of DR. Given that ischemic changes in the retinal capillaries initially occur in the mid-peripheral retina, a wider field of view is necessary for accurate assessment of DR severity. Tan et al. compared the diagnostic accuracy of DR using a cropped 12×12 mm^2^ OCTA image into a central 6×6 mm^2^ field and peripheral square annulus region [36]. They reported that capillary dropout density in the peripheral square annulus was the most effective parameter for distinguishing between mild NPDR and no DR. Our group also previously described that the microvascular parameters from 10×10 mm^2^ OCTA images were more sensitive than 3×3 mm^2^ and 6×6 mm^2^ images for determining DR severity [16]. Consistent with previous studies, our research confirmed that NPA from larger field of view OCTA scans may be more valuable in predicting microalbuminuria in DR patients.

However, it is important to note that OCTA imaging involves a complex set of image-processing steps and is susceptible to various artifacts. Most earlier studies reported that at least one artifact exists in more than 95% of OCTA images [37]. Among these, low-signal artifacts confounded the identification of NPA, and consequently, quantitative measurements become clinically unreliable. Factors contributing to low OCT signals include media opacity, vignetting, ocular aberrations, and backscattering [38]. Wide-field OCTA imaging, such as the 15×9 mm^2^ or 12×12 mm^2^ field of view provided by SS-OCTA, is more prone to artifacts due to longer image acquisition times, patient cooperation challenges, and increased motion artifacts. Wide-field imaging may produce low-signal artifacts more frequently, especially in shadowed areas or peripheral regions that are out of focus due to retinal curvature [37]. To detect low OCT signal artifacts, a reliable strategy is to compare OCTA and OCT en face images simultaneously [37, 39]. Since OCTA is derived from structural OCT, which performs repeated B-scans, corresponding structural images are available alongside OCTA images [40]. True NPA should only appear in the OCTA signal, whereas artifacts affecting the structural and angiographic signals cause signal loss. Recognizing, minimizing, or controlling such artifacts is crucial to avoid misinterpretation in clinical practice.

The main limitation of our study is that we used three cropped squares of different sizes from a 12×12 mm^2^ OCTA image for analyzing OCTA parameters. The lower lateral resolution of larger scans (cropped from 12×12 mm^2^) compared to the original 3×3 mm^2^ scan may underestimate NPA represented with small-vessel dropout. Additionally, we used spot urine albumin-to-creatinine ratio (ACR) instead of the gold standard 24-hour urine collection for quantifying urinary albumin due to the retrospective study design. Lastly, we did not consider various factors that can affect albumin levels in random urine samples, such as antihypertensive medication, hydration status, and environmental factors.

This study investigated the correlation between wide-field SS-OCTA parameters and albuminuria in patients with different grades of DR. Our findings suggest that DR with a larger NPA is associated with an increased risk of nephropathy, which can be explained by mid-peripheral capillary nonperfusion in the presence of clinical albuminuria. In conclusion, we emphasize the importance of mid-peripheral capillary nonperfusion in patients with clinically visible DR and recommend early screening for diabetic nephropathy and monitoring of kidney function.

## Supporting information

S1 Data (PDF)
