# Supplementary material for: Association between albuminuria and retinal microvascular parameters measured with swept-source optical coherence tomography angiography in patients with diabetic retinopathy
Source: PLoS One. 2024 Mar 6;19(3):e0295768. doi: 10.1371/journal.pone.0295768 (PMC10917288; doi:10.1371/journal.pone.0295768)
Supplement: S1 Data — (PDF) [file pone.0295768.s001.pdf]

Sex	age	duratic	DR gra	BCVA	HbA1c	MAP	Insuli	HTN	microalbu	eGFR
2	59	10	3	1	8.3	96.66667	2	2	0.1	82.26
2	59	10	3	1	8.3	96.66667	2	2	0.1	82.26
2	54	3	2	1	7	93.66667	2	2	2.1	115
2	47	2	2	0.9	7.2	112.3333	2	1	2.6	92.84
1	39	1	2	1	6.7	94.66667	2	2	3.2	80
1	59	2	4	1	7.2	97	2	2	3.5	116
1	59	2	4	1	7.2	97	2	2	3.5	116
1	56	4	1	1	5.9	100.6667	2	1	3.7	96
1	56	4	1	1	5.9	100.6667	2	1	3.7	96
1	49	17	5	0.9	6.6	100.6667	1	2	3.9	109
1	55	12	3	1	8	96.33333	1	2	4.1	110
1	37	1	3	1	5.3	95.66667	2	1	4.1	124
1	37	1	4	1	5.3	95.66667	2	1	4.1	124
1	69	10	1	1	9.6	83	2	2	4.8	128
1	43	1	3	1	8	106.6667	1	1	4.8	90
1	43	1	3	1	8	106.6667	1	1	4.8	90
1	60	1	1	1	6.8	89	2	2	4.9	120
1	45	1	3	1	6.4	92.33333	2	1	5	103
1	45	1	3	1	6.4	92.33333	2	1	5	103
2	66	10	1	1	7	81	2	1	5.1	72.2
1	65	3	2	1	7.8	72.33333	2	2	5.6	84.73
1	56	8	3	0.7	6.2	101.3333	2	1	5.8	108
1	56	8	4	1	6.2	101.3333	2	1	5.8	108
2	59	7	3	1	7.2	61	2	1	5.9	169
2	59	7	4	1	7.2	61	2	1	5.9	169
1	51	13	2		8.5	92	2	2	6.7	96
1	51	13	2		8.5	92	2	2	6.7	96
1	53	4	4	1	6.4	96	2	1	6.7	115.6
1	53	4	5	1	6.4	96	2	1	6.7	115.6
2	59	6	1	1	6	70.66667	2	2	6.9	91
2	69	30	1	1	7.9	86.66667	2	2	7	107.59
2	54	4	4	0.9	8.1	90.33333	2	2	7.1	118
2	68	15	1	1	7.3	89.66667	2	1	7.2	66.29
2	68	15	2	1	7.3	89.66667	2	1	7.2	66.29
1	63	14	1	1	7.5	88	2	2	7.4	115.97
1	45	5	2	0.9	8.2	97	2	2	7.6	104.3
1	45	5	3	1	8.2	97	2	2	7.6	104.3
2	63	20	2	1	7.1	94.66667	2	1	7.8	173
2	76	20	2	0.6	7.9	95	2	1	7.8	69.23
2	76	20	2	0.8	7.9	95	2	1	7.8	69.23
1	41	1	4	0.8	7.1	106	2	2	8.3	128
1	41	1	5	1	7.1	106	2	2	8.3	128

1	47	3	3	1	6.9	99	1	2	8.7	93.94
1	47	3	3	1	6.9	99	1	2	8.7	93.94
1	35	1	4	1	5	102	2	1	8.8	176
1	35	1	4	1	5	102	2	1	8.8	176
2	71	7	3	1	9	88	2	1	10	60
2	63	13	4	1	7.4	90	2	1	10.2	55.14
2	63	13	4	1	7.4	90	2	1	10.2	55.14
1	75	19	1	1	7	76.66667	2	1	10.5	78.5
1	75	19	1	1	7	76.66667	2	1	10.5	78.5
2	76	10	2	1	6.7	97.33333	2	2	10.7	97.81
1	45	1	4	1	5.4	74	2	2	10.9	101.55
1	45	1	4	1	5.4	74	2	2	10.9	101.55
2	53	3	1	0.7	6.4	91.33333	2	2	11	101.9
2	53	3	1	1	6.4	91.33333	2	2	11	101.9
2	70	8	2	0.7	6.8	102	2	1	11.6	85
2	75	12	2	0.7	6.9	102	2	1	12	50.25
2	75	12	2	0.7	6.9	102	2	1	12	50.25
1	58	20	3	1	7.9	78.33333	2	1	12.4	118
1	57	20	3	0.7	7.8	81	2	1	12.6	120
1	57	20	3	0.9	7.8	81	2	1	12.6	121
2	70	18	4	1	7.4	115.6667	1	1	12.6	95
2	67	1	1	1	6.5	87	2	2	14.1	145
1	51	20	5	1	7.2	98.33333	1	2	14.6	93
1	51	20	5	1	7.2	98.33333	1	2	14.6	93
1	46	6	4	1	6.6	74	2	2	14.9	157
1	40	7	4	1	5.6	96.66667	2	1	15.3	138
1	40	7	4	1	5.6	96.66667	2	1	15.3	138
2	67	15	3	1	10.1	99.33333	2	2	15.5	106.16
2	67	15	3	1	10.1	99.33333	2	2	15.5	106.16
1	66	11	5	1	8.2	88.33333	2	1	16.2	155
1	63	4	2	1	6.6	86.66667	2	1	16.5	88.51
1	61	13	1	1	8.5	79.33333	2	2	16.9	128
1	61	13	2	1	8.5	79.33333	2	2	16.9	128
2	60	15	2	1	5.9	73.33333	2	1	17.1	122.6
2	60	15	2	1	5.9	73.33333	2	1	17.1	122.6
1	65	10	2	1	8.9	71.33333	2	2	17.3	134.12
1	60	1	1	1	7	109.3333	2	1	17.6	135.8
1	60	1	2	1	7	109.3333	2	1	17.6	135.8
2	58	1	3	1	6	84	1	2	18	169
2	58	1	4	1	6	84	1	2	18	169
2	69	20	1	0.9	6.7	94	2	2	18.1	98
1	53	1	4	1	11.4	95.33333	1	2	20.8	127
1	53	1	4	1	11.4	95.33333	1	2	20.8	127

2	58	10	3	0.9	7.3	79.66667	2	1	22.4	68.71
2	58	10	3	0.9	7.3	79.66667	2	1	22.4	68.71
1	64	15	3	0.9	6.7	98	2	1	23.1	77.45
1	64	15	3	0.9	6.7	98	2	1	23.1	77.45
1	46	1	1	1	6.6	111	2	1	23.6	31.9
1	46	1	1	1	6.6	111	2	1	23.6	31.9
2	65	10	4		6.4	92.33333	1	2	23.7	86.82
2	68	6	4	1	7.1	91.66667	2	1	27.8	102
2	68	6	4	0.9	7.1	91.66667	2	1	27.8	102
2	69	20	3	1	7.7	87	2	1	28.4	78.19
2	69	20	3	1	7.7	87	2	1	28.4	78.19
1	61	15	3	1	8.8	100.6667	2	1	31.5	85.86
1	61	15	3	1	8.8	100.6667	2	1	31.5	85.86
1	64	1	1	1	5.8	95.33333	2	1	32.6	113
1	64	1	1	0.9	5.8	95.33333	2	1	32.6	113
2	67	15	1	1	6	83.66667	2	1	33.2	100.65
2	67	15	1	1	6	83.66667	2	1	33.2	100.65
1	49	1	5	1	8.5	105	2	2	40	129
1	49	1	5	1	8.5	105	2	2	40	129
2	65	15	5	0.5	7.5	87	2	1	47.7	80.35
1	52	1	5	0.9	12.4	143	1	1	72	89.77
2	48	4	1	1	6.4	88	2	1	88	100
2	63	1	5	0.5	5.6	91	2	1	96.6	88.52
2	65	15	3	1	7.9	91	2	2	100	118
2	65	15	3	1	7.9	91	2	2	100	118
1	50	10	4	1	12	86.66667	2	2	100	93
1	64	20	4	1	8.9	108	2	1	103.9	95.68
2	49	13	5	0.5	7.8	90.66667	1	2	106	149
1	52	1	4	1	5.8	84.66667	2	1	119.4	49.14
1	52	1	4	1	5.8	84.66667	2	1	119.4	49.14
2	73	20	4	1	6.5	102.6667	2	1	130	47
2	73	20	4	1	6.5	102.6667	2	1	130	47
1	47	1	4	1	6.9	92.66667	2	2	141	107
1	51	10	4	0.9	7.1	127	1	1	162	37.28
1	51	10	4	0.9	7.1	127	1	1	162	37.28
1	53	1	4	0.8	7.3	92	1	1	196	99.1
1	53	1	4	0.9	7.3	92	1	1	196	99.1
2	71	8	3	1	7.1	104	2	1	220	63
2	71	8	3	1	7.1	104	2	1	220	63
1	38	20	4	1	10.9	96	1	2	300	128
1	38	20	4	1	10.9	96	1	2	300	128
1	53	10	3	1	9.7	88.33333	2	1	304.5	117.86
1	53	10	3	1	9.7	88.33333	2	1	304.5	117.86

1	48	1	5	0.8	7.4	118.6667	2	1	328	72
1	48	1	5	0.8	7.4	118.6667	2	1	328	72
2	50	20	4	0.4	8.1	72	1	2	473	83.23
2	44	4	4	0.8	7	88.66667	2	1	668	29.67
1	39	1	4	0.9	8.9	83.66667	1	1	677	67.25
1	39	1	4	0.8	8.9	83.66667	1	1	677	67.25
1	41	3	1	1	10	102.6667	2	1	725	73
1	41	3	1	1	10	102.6667	2	1	725	73
1	65	20	3	0.8	7.7	82.33333	2	2	800	69.38
1	65	20	3	0.7	7.7	82.33333	2	2	800	69.38
1	67	25	5	0.8	7.9	101.6667	2	2	884	71.13
2	52	7	3	1	6.3	87.33333	2	1	1125	23.7
2	75	25	4	1	7.2	86.66667	1	2	1147	38
1	50	10	4	0.6	14	98	2	2	1262	104.47
1	58	10	1	1	6.5	90.66667	2	1	1293	80
1	49	2	4	0.9	6.2	89	2	2	2458	15.35
1	69	10	5		7.5	107	2	1	2600	20
2	46	10	3	1	13.9	83.33333	2	2	3776	254
2	46	10	3	1	13.9	83.33333	2	2	3776	254
1	63	12	3	0.9	6.9	90.33333	2	1	4232	17
2	65	13	2	1	7.3	96	2	1	304.5	133
2	65	13	2	1	7.3	96	2	1	304.5	133
2	54	4	4	0.9	8.1	90.33333	2	2	7.1	118
2	68	15	1	1	7.3	89.66667	2	1	7.2	66.29
2	68	15	2	1	7.3	89.66667	2	1	7.2	66.29
2	49	13	5	0.5	7.8	90.66667	1	2	106	149
2	58	10	3	0.9	7.3	79.66667	2	1	22.4	68.71
2	58	10	3	0.9	7.3	79.66667	2	1	22.4	68.71
2	70	18	4	1	7.4	115.6667	1	1	12.6	95
1	59	1	5	0.6	6.8	83.33333	2	1	884	103.9
1	52	1	4	1	5.8	84.66667	2	1	119.4	49.14
1	52	1	4	1	5.8	84.66667	2	1	119.4	49.14
1	49	2	4	0.9	6.2	89	2	2	2458	15.35
1	65	10	2	1	8.9	71.33333	2	2	17.3	134.12
2	50	20	4	0.4	8.1	72	1	2	473	83.23
1	60	1	1	1	6.8	89	2	2	4.9	120
1	52	3	5	1	5.6	115.3333	2	2	300	97.26
2	79	20	3	1	6.4	100	2	1	300	42.17
1	63	4	2	1	6.6	86.66667	2	1	16.5	88.51
1	62	18	3	0.9	8.5	92.66667	2	2	196	116
2	64	1	5	0.8	6.6	73	2	2	220	105.46
1	53	10	5	1	10.2	92.33333	2	2	2600	62
1	53	10	5	1	10.2	92.33333	2	2	17.3	62

1	41	1	4	0.8	7.1	106	2	2	8.3	128
1	41	1	5	1	7.1	106	2	2	8.3	128
1	60	1	1	1	7	109.3333	2	1	17.6	135.8
1	60	1	2	1	7	109.3333	2	1	17.6	135.8
1	38	1	4	1	5.8	96.66667	2	1	3776	147.25
1	38	1	4	1	5.8	96.66667	2	1	3776	147.25
1	46	1	1	1	6.6	111	2	1	23.6	31.9
1	46	1	1	1	6.6	111	2	1	23.6	31.9
1	35	1	4	1	5	102	2	1	8.8	176
1	38			1		82.33333		1		
2	63			1		82.33333		2		
1	45			1		102		1		
1	45			1		102		2		
1	74			1		86.66667		2		
1	74			1		79.33333		1		
1	41			1		79.33333		1		
1	41			1		90.66667		1		
1	37			1		88		2		
1	37			1		113.3333		1		
1	56			1		104		1		
1	56			1		100.6667		2		
1	45			0.9		100.6667		1		
1	45			0.9		97.33333		1		
2	58			1		88.33333		1		
2	58			1		93		1		
1	65			0.9		88		1		
1	48			1		101		1		
1	48			1		106		2		
1	50			0.9		100.6667		2		
1	51			1		98		2		
1	51			0.9		102.6667		2		
1	56			1		102.6667		2		
1	56			1		97		1		
2	64			0.8		84.66667		1		
2	64			1		84.66667		2		
1	53			1		89		2		
1	53			1		79.66667		2		
1	72			1		115.6667		2		
2	59			0.8		96.66667		2		
2	59			1		96.66667		2		
1	63			1		90.33333		1		
2	68			1		91.66667		1		
2	68			0.9		91.66667		1		

2	48	1	96	1
2	48	1	96	1
1	45	0.8	110	1
2	65	1	91	2
2	65	1	91	2
1	69	1	83	2
2	52	1	87.33333	1
1	67	0.8	101.6667	2
2	67	1	99.33333	2
2	67	1	99.33333	2
1	65	1	72.33333	2
2	66	1	81	1
2	53	0.7	91.33333	2
2	53	1	91.33333	2
2	72	1	96.66667	1
1	58	1	78.33333	1

FAZ area	FAZ circul	NPA_rect(	NPA_rect(	NPA_rect(	VD_rec_m	VD_rec_m	VD_rec_m_10
0.430166	0.653	0.3273	0.4064	5.5744	44.1669	53.9761	50.0947
0.444732	0.674	0.2261	0.2808	4.1858	47.2663	55.9165	50.3612
0.261844	0.689	0.218	0.2689	2.0546	45.5203	53.8105	49.4339
0.49054	0.736	0.2988	0.3717	5.3157	46.3395	55.6588	51.5485
0.404606	0.475	0	0	3.0408	54.9231	62.1841	54.311
0.347056	0.654	0.2726	0.5019	14.5536	52.0132	51.8511	48.8117
0.380891	0.484	0.3537	0.5295	14.5115	53.5956	53.9667	49.0119
0.564071	0.767	0.2733	0.3398	2.4013	40.5358	48.9334	46.2632
0.451083	0.663	0.0124	0.0154	1.4937	45.2849	48.918	44.8887
0.410065	0.564	0.4682	0.5986	14.3665	45.4959	54.0463	51.2374
0.49417	0.472	0.2093	0.2921	6.8246	42.6017	51.9339	49.7082
0.577881	0.444	0.4549	0.567	4.844	41.5085	53.2806	52.4099
0.495363	0.663	0.6459	0.8005	5.2333	42.3916	52.8582	50.8024
0.229503	0.585	0	0	3.2193	47.8696	55.7349	49.3856
0.594584	0.577	0.7853	0.9724	8.5315	40.4867	53.2679	49.8091
0.4975	0.725	0.5826	0.7175	4.1978	40.9388	52.1312	50.4291
0.389345	0.606	0.2578	0.3193	2.371	50.2359	57.7405	53.5811
0.456233	0.544	0.3376	0.4179	3.4912	48.2833	54.7459	49.0292
0.495707	0.584	0.3333	0.4106	3.9724	48.0776	52.1541	46.7427
0.405979	0.839	0.2844	0.3512	3.3054	47.8901	58.5165	50.9352
0.258488	0.536	0.1781	0.2208	2.8469	49.5005	54.2615	50.6165
0.911385	0.592	0.9406	1.4468	17.4828	45.7558	52.9549	51.0591
0.779609	0.316	0.5298	1.2615	26.2512	49.353	53.3274	50.0743
0.25036	0.511	0.2144	0.3101	11.7911	49.0418	55.3324	50.637
0.221357	0.679	0.2047	0.287	8.9438	53.4575	58.5755	51.6142
0.2234	0.535	0.2536	0.3193	3.7886	46.1136	53.7037	47.4249
0.247973	0.537	0.3182	0.3934	3.7584	45.9465	52.7172	47.0116
0.302579	0.685	0.3809	0.8015	21.2217	42.3273	48.6553	46.6567
0.353142	0.408	0.3329	1.1453	21.3907	44.0972	48.2843	45.3439
0.36733	0.765	0.1916	0.2387	3.7083	52.671	56.6421	49.5256
0.527781	0.573	0	0	2.4342	48.2298	54.0471	50.1705
0.449332	0.464	0.2827	0.3495	9.2186	51.2288	59.9501	51.6903
0.695005	0.562	0	0	2.3268	55.3145	60.742	52.0991
0.65827	0.676	0.4648	0.576	7.5111	53.642	60.5539	52.0288
0.460061	0.551	0.3623	0.5148	14.4703	48.2524	53.299	46.3406
0.252325	0.59	0.0227	0.0281	3.0796	52.4622	56.3079	51.9712
0.252025	0.676	0.0015	0.0018	2.3789	57.4184	57.9077	51.0825
0.401044	0.713	0.4682	0.5827	8.0471	45.1286	55.2744	49.2793
0.333529	0.703	0	0	0.7591	52.1966	53.4125	47.7035
0.411738	0.498	0.0054	0.0067	1.123	51.4651	54.0698	50.2619
0.315814	0.495	0.3134	0.4065	11.9224	41.0744	50.6969	47.8527
0.289713	0.583	1.0777	1.7112	24.2428	45.2545	52.5959	47.7358

0.302785	0.663	0.2705	0.4228	8.7701	52.8591	58.3191	49.3125
0.314638	0.662	0.3062	0.4357	9.0935	51.1182	56.5207	49.3603
0.432037	0.66	0.8556	2.017	16.8728	36.9798	46.3293	47.7376
0.023586	0.64	0.5075	0.742	13.846	39.9768	50.1746	48.5435
0.594309	0.441	0.2884	0.3569	2.845	46.5286	56.1681	49.4098
0.45013	0.45	0.9189	2.3889	25.6207	36.8844	49.5127	48.8478
0.686002	0.472	0.5949	0.8331	17.2433	47.5614	54.9454	49.6994
0.60672	0.43	0	0	0.4212	48.978	54.0365	49.8479
0.210637	0.698	0	0	0.3218	46.179	50.8231	48.804
0.468258	0.609	0.0061	0.0076	2.0546	51.3959	53.061	44.3512
0.305566	0.49	0.7919	1.3726	24.2752	42.6894	50.4553	46.6009
0.460344	0.271	0.9441	1.4382	19.7536	39.2392	49.2824	46.1514
0.422218	0.595	0.2329	0.2871	2.1753	54.9122	57.4069	50.4084
0.43208	0.591	0.3626	0.4481	2.1462	51.4271	58.1984	53.9107
0.410545	0.759	0.0104	0.0129	6.1686	50.3428	59.7892	51.4349
0.28537	0.72	0	0	2.8097	53.6946	58.6882	49.5777
0.352661	0.77	0.2389	0.2958	3.1713	34.2638	45.1499	46.0251
0.359296	0.451	0.3425	0.4932	18.959	46.6032	54.5979	49.1625
0.54039	0.392	0.7173	1.2423	17.571	41.9152	38.7371	39.3396
0.464035	0.418	0.4972	0.726	12.5438	35.3886	44.5256	45.474
0.403473	0.63	0.326	0.6438	15.3677	41.0809	51.8093	47.5196
0.385757	0.722	0.0034	0.0042	0.1307	38.7175	53.8943	49.4577
0.358978	0.666	0.3405	0.4367	9.3526	42.3174	52.0678	48.9528
0.360352	0.411	0.3786	1.2646	21.646	48.6895	53.1256	48.025
0.352489	0.631	0.5035	1.277	19.2867	46.0367	51.9123	48.0703
0.459666	0.312	0.3099	0.4268	13.4606	52.5519	54.7701	49.2997
0.306973	0.379	0.2338	0.3307	8.8751	48.6625	51.0596	48.1222
0.306227	0.796	0	0	1.2168	50.827	56.7671	52.8072
0.393851	0.517	0	0	2.1147	52.5677	58.0102	51.2683
0.428493	0.55	0.4309	0.6187	31.6476	43.9855	46.4261	43.7236
0.330397	0.615	0.245	0.3045	2.1481	37.8194	51.4467	50.2743
0.327976	0.565	0.058	0.0719	2.476	47.7928	53.5208	50.0875
0.360472	0.591	0.0977	0.1212	1.848	47.1356	52.3739	49.1535
0.293009	0.469	0.2779	0.348	4.4361	46.2236	51.948	48.7568
0.308347	0.522	0.1059	0.1317	4.1708	49.1371	54.4719	48.8932
0.416468	0.68	0.4921	0.6062	7.787	41.724	54.8071	50.4628
0.232043	0.654	0.2407	0.2968	3.3523	52.0797	57.4894	50.2735
0.254497	0.516	0.1031	0.1267	0.3847	53.2174	57.6191	52.6296
0.41597	0.614	0.3848	0.4832	3.5303	46.3044	52.0401	52.044
0.452044	0.562	0.4947	0.7184	7.9441	53.2277	55.1542	55.7394
0.475373	0.613	0.012	0.0149	2.4166	55.9024	57.9474	49.5992
0.416639	0.578	0.2844	0.4423	13.559	51.4079	54.9459	48.6817
0.362926	0.621	0.4851	0.6499	9.6796	44.3064	53.7128	49.9121

0.43105	0.522	0.4644	1.1565	34.9035	50.0976	54.7818	50.7258
0.414528	0.543	1.2218	2.301	23.0237	44.2709	54.086	49.5148
0.467606	0.657	0.7613	0.9879	12.9287	44.3392	48.4208	47.6169
0.46582	0.58	0.3278	0.4827	14.4498	46.3508	46.7561	47.6153
0.195265	0.738	0.1242	0.1546	3.2691	50.0611	56.1793	50.1398
0.208277	0.537	0.084	0.1043	1.9248	50.4182	57.5908	52.1146
0.617964	0.372	0.6863	1.1929	22.4539	38.1837	48.6818	47.0711
0.297978	0.686	0.3839	1.4975	20.1767	44.9297	47.0639	43.3364
0.316681	0.735	0.3345	0.5399	15.0747	43.3062	46.7022	44.6282
0.355322	0.724	0.2955	0.3897	8.0892	53.2016	54.6801	49.3246
0.320037	0.792	0.0414	0.0513	12.8496	59.1437	57.718	50.6763
0.36333	0.505	0.1864	0.2434	5.7484	55.5127	55.4249	49.7537
0.289455	0.671	0.3041	0.3778	4.5859	46.829	51.271	48.8493
0.321299	0.843	0.1871	0.2306	1.838	52.8938	61.5835	59.6702
0.33316	0.716	0.1759	0.2184	1.4961	48.8937	57.1085	57.0441
0.335778	0.746	0.0057	0.0071	1.0629	45.4508	55.4569	49.511
0.273208	0.768	0.0089	0.011	2.086	46.1886	52.3354	48.2172
0.329178	0.672	0.3061	0.4805	8.622	44.3295	54.336	48.2328
0.3815	0.545	0.4035	0.5169	12.7829	41.8163	50.6548	45.4856
0.648125	0.419	0.7832	1.8519	27.4708	40.6335	49.8701	45.4699
0.400829	0.371	0.6059	1.3481	32.9092	46.1059	51.5668	46.2962
0.576241	0.718	0.5618	0.6943	6.3813	48.8847	56.2222	50.906
1.859239	0.41	3.1961	13.4397	110.3918	51.1662	51.4696	45.0064
0.521439	0.82	0.3617	0.4457	5.9327	50.5472	55.4404	49.7251
0.388641	0.858	0.436	0.5378	7.9229	47.724	55.2656	49.9845
0.29621	0.512	0.9335	2.7994	44.3164	42.2079	48.5915	43.5881
0.438354	0.328	0.3607	0.5188	15.5113	53.8069	57.7845	50.957
0.436947	0.466	0.7227	1.8148	25.0436	44.3288	49.8635	46.0107
0.395594	0.48	0.5152	0.8059	16.6886	43.3703	51.3081	45.7558
0.669342	0.4	0.7137	1.919	29.7648	42.9665	47.7063	44.6998
0.470223	0.456	0.3358	0.4778	10.6481	54.3456	60.2508	50.2459
0.408142	0.728	0.3869	0.5383	18.6955	45.5507	54.2175	47.9208
0.271637	0.565	0.3864	0.5919	12.3509	43.6151	53.1631	49.4173
0.594266	0.426	1.6654	4.4983	45.5416	45.4703	51.5102	48.7842
0.482815	0.512	2.6086	4.9972	37.2551	38.8286	49.0971	48.0214
0.403404	0.611	0.4294	0.9868	24.1217	48.8419	52.6937	47.1503
0.393105	0.649	0.7555	1.7252	21.0294	41.7839	50.2677	45.7925
0.306862	0.789	0.2611	0.3323	6.4584	51.0401	48.584	44.8659
0.332474	0.811	0.3177	0.437	9.3889	43.1741	47.4359	45.0927
0.385191	0.614	0.5245	2.3979	27.3328	41.0621	48.2274	43.8472
0.3457	0.593	0.9658	2.9199	43.4651	42.3026	49.9821	44.4189
0.148659	0.522	0.1243	0.1538	4.6935	33.8734	47.2737	44.6715
0.221889	0.348	0.244	0.3055	2.6482	32.3607	43.8059	43.4554

0.336199	0.43	3.1075	7.0334	65.4883	38.8595	40.3064	37.5235
0.515061	0.288	1.4446	5.6384	66.1333	38.8595	42.9078	41.7188
0.419249	0.539	0.4431	2.5256	23.5325	40.3243	47.0192	45.9156
0.328448	0.351	0.2203	0.2736	7.031	38.6622	52.9894	51.0606
0.205762	0.634	0.4284	1.0474	19.193	35.1969	49.0771	46.7406
0.232464	0.482	0.4729	0.7905	14.9792	43.9845	48.2475	47.4493
0.287044	0.608	0	0	1.2387	65.8516	68.2952	52.6233
0.372402	0.667	0	0.0047	2.556	59.3673	62.8876	50.7457
0.467056	0.546	0.3753	0.4747	6.0633	41.4353	48.6145	47.7516
0.352017	0.716	0.3327	0.5269	8.9359	45.9394	47.5935	46.1351
0.428862	0.32	0.522	2.0574	38.0669	45.5123	54.6098	49.4826
0.504702	0.485	0.2973	0.3839	8.031	50.1635	58.7198	50.2481
0.26471	0.521	0.064	0.0839	13.166	49.8738	50.9898	39.28
0.267054	0.62	0.3547	2.3073	23.4389	46.1198	49.4927	47.6993
0.193171	0.662	0.1228	0.1517	4.5275	42.5475	52.6613	50.5234
0.479424	0.749	0.4278	0.7903	25.8911	57.8332	59.9353	49.7841
0.406709	0.197	0.6814	1.0606	15.0465	38.9353	48.7544	45.0433
0.391474	0.477	0.1166	0.1693	3.6472	46.2229	55.4379	51.3947
0.301927	0.731	0.1656	0.2063	3.1668	43.362	53.7657	50.3717
0.508573	0.804	0.4773	0.5903	4.3391	46.0037	52.9687	49.4505
0.342722	0.473	0.1081	0.1339	1.9418	53.2332	53.8084	49.4008
0.342018	0.496	0	0	2.4407	56.788	57.5622	50.8973
0.449332	0.464	0.2827	0.3495	9.2186	51.2288	59.9501	51.6903
0.695005	0.562	0	0	2.3268	55.3145	60.742	52.0991
0.65827	0.676	0.4648	0.576	7.5111	53.642	60.5539	52.0288
0.436947	0.466	0.7227	1.8148	25.0436	44.3288	49.8635	46.0107
0.43105	0.522	0.4644	1.1565	34.9035	50.0976	54.7818	50.7258
0.414528	0.543	1.2218	2.301	23.0237	44.2709	54.086	49.5148
0.403473	0.63	0.326	0.6438	15.3677	41.0809	51.8093	47.5196
0.62168	0.483	0.6006	3.118	34.3164	44.9242	46.7175	43.4669
0.395594	0.48	0.5152	0.8059	16.6886	43.3703	51.3081	45.7558
0.669342	0.4	0.7137	1.919	29.7648	42.9665	47.7063	44.6998
0.479424	0.749	0.4278	0.7903	25.8911	57.8332	59.9353	49.7841
0.416468	0.68	0.4921	0.6062	7.787	41.724	54.8071	50.4628
0.419249	0.539	0.4431	2.5256	23.5325	40.3243	47.0192	45.9156
0.389345	0.606	0.2578	0.3193	2.371	50.2359	57.7405	53.5811
0.404709	0.6	2.6914	5.7149	31.1701	28.7466	40.9413	44.3637
0.417206	0.7	0.399	0.5436	16.7264	56.8065	58.1459	48.5741
0.330397	0.615	0.245	0.3045	2.1481	37.8194	51.4467	50.2743
0.292949	0.516	0.32	2.6235	41.4615	50.4807	51.2997	45.7294
0.416382	0.6	0.9849	2.6708	32.8851	32.5141	40.7354	41.7203
0.417129	0.646	2.0917	4.9481	39.8492	32.5847	43.6404	42.7421
0.385963	0.574	1.0621	2.9945	30.1587	37.1912	44.4207	42.9356

0.315814	0.495	0.3134	0.4065	11.9224	41.0744	50.6969	47.8527
0.289713	0.583	1.0777	1.7112	24.2428	45.2545	52.5959	47.7358
0.232043	0.654	0.2407	0.2968	3.3523	52.0797	57.4894	50.2735
0.254497	0.516	0.1031	0.1267	0.3847	53.2174	57.6191	52.6296
0.428141	0.383	0.2979	1.1392	23.2562	53.6607	53.8989	49.5972
0.330766	0.403	0.3706	1.5811	34.439	51.7353	56.1505	48.5407
0.195265	0.738	0.1242	0.1546	3.2691	50.0611	56.1793	50.1398
0.208277	0.537	0.084	0.1043	1.9248	50.4182	57.5908	52.1146
0.432037	0.66	0.8556	2.017	16.8728	36.9798	46.3293	47.7376
0.467056	0.546	0.3753	0.4747	6.0633	41.4353	48.6145	47.7516
0.352017	0.716	0.3327	0.5269	8.9359	45.9394	47.5935	46.1351
0.28537	0.72	0	0	2.8097	53.6946	58.6882	49.5777
0.352661	0.77	0.2389	0.2958	3.1713	34.2638	45.1499	46.0251
0.29621	0.512	0.9335	2.7994	44.3164	42.2079	48.5915	43.5881
0.327976	0.565	0.058	0.0719	2.476	47.7928	53.5208	50.0875
0.360472	0.591	0.0977	0.1212	1.848	47.1356	52.3739	49.1535
0.193171	0.662	0.1228	0.1517	4.5275	42.5475	52.6613	50.5234
0.460061	0.551	0.3623	0.5148	14.4703	48.2524	53.299	46.3406
0.564071	0.767	0.1066	0.1326	3.576	52.985	56.5191	50.3612
0.291026	0.851	0	0	3.1645	57.7629	63.4377	51.5445
0.564071	0.767	0.2733	0.3398	2.4013	40.5358	48.9334	46.2632
0.451083	0.663	0.0124	0.0154	1.4937	45.2849	48.918	44.8887
0.468258	0.609	0.0061	0.0076	2.0546	51.3959	53.061	44.3512
0.428493	0.55	0.4309	0.6187	31.6476	43.9855	46.4261	43.7236
0.534657	0.587	0.4952	0.6985	6.0458	34.78	46.4667	45.2183
0.594309	0.441	0.2884	0.3569	2.845	46.5286	56.1681	49.4098
0.238678	0.674	0	0.0251	9.1616	49.5756	54.3285	46.5903
0.454834	0.733	0	0	2.5638	36.8306	49.5517	48.7115
0.410065	0.564	0.4682	0.5986	14.3665	45.4959	54.0463	51.2374
0.267054	0.62	0.3547	2.3073	23.4389	46.1198	49.4927	47.6993
0.470223	0.456	0.3358	0.4778	10.6481	54.3456	60.2508	50.2459
0.408142	0.728	0.3869	0.5383	18.6955	45.5507	54.2175	47.9208
0.347056	0.654	0.2726	0.5019	14.5536	52.0132	51.8511	48.8117
0.380891	0.484	0.3537	0.5295	14.5115	53.5956	53.9667	49.0119
0.49417	0.472	0.2093	0.2921	6.8246	42.6017	51.9339	49.7082
0.648125	0.419	0.7832	1.8519	27.4708	40.6335	49.8701	45.4699
0.438354	0.328	0.3607	0.5188	15.5113	53.8069	57.7845	50.957
0.148659	0.522	0.1243	0.1538	4.6935	33.8734	47.2737	44.6715
0.221889	0.348	0.244	0.3055	2.6482	32.3607	43.8059	43.4554
0.640769	0.76	0.4544	0.5632	4.8781	44.2174	55.7573	50.5393
0.402512	0.68	0.2835	0.381	5.0361	45.0163	55.9271	50.5872
0.329178	0.672	0.3061	0.4805	8.622	44.3295	54.336	48.2328
0.3815	0.545	0.4035	0.5169	12.7829	41.8163	50.6548	45.4856

0.504702	0.485	0.2973	0.3839	8.031	50.1635	58.7198	50.2481
0.428862	0.32	0.522	2.0574	38.0669	45.5123	54.6098	49.4826
0.306227	0.796	0	0	1.2168	50.827	56.7671	52.8072
0.393851	0.517	0	0	2.1147	52.5677	58.0102	51.2683
0.258488	0.536	0.1781	0.2208	2.8469	49.5005	54.2615	50.6165
0.405979	0.839	0.2844	0.3512	3.3054	47.8901	58.5165	50.9352
0.422218	0.595	0.2329	0.2871	2.1753	54.9122	57.4069	50.4084
0.43208	0.591	0.3626	0.4481	2.1462	51.4271	58.1984	53.9107
0.445607	0.473	0.4726	0.6079	11.0744	45.8631	51.0791	44.1878
0.359296	0.451	0.3425	0.4932	18.959	46.6032	54.5979	49.1625
0.193171	0.662	0.1228	0.1517	4.5275	42.5475	52.6613	50.5234
0.460061	0.551	0.3623	0.5148	14.4703	48.2524	53.299	46.3406
0.564071	0.767	0.1066	0.1326	3.576	52.985	56.5191	50.3612
0.352661	0.77	0.2389	0.2958	3.1713	34.2638	45.1499	46.0251
0.29621	0.512	0.9335	2.7994	44.3164	42.2079	48.5915	43.5881
0.193171	0.662	0.1228	0.1517	4.5275	42.5475	52.6613	50.5234
